# Epithelial to Mesenchymal Transition: Key Regulator of Pancreatic Ductal Adenocarcinoma Progression and Chemoresistance

**DOI:** 10.3390/cancers13215532

**Published:** 2021-11-04

**Authors:** Kostas Palamaris, Evangelos Felekouras, Stratigoula Sakellariou

**Affiliations:** 1 1ST Department of Pathology, Medical School, National and Kapodistrian University of Athens, 11527 Athens, Greece; kpalamaris@yahoo.gr; 2 1ST Department of Surgery, Medical School, National and Kapodistrian University of Athens, 11527 Athens, Greece; felek@med.uoa.gr

**Keywords:** pancreatic ductal adenocarcinoma, epithelial to mesenchymal transition, cancer stem cells, intratumor heterogeneity, tumor microenvironment, chemoresistance

## Abstract

**Simple Summary:**

Pancreatic ductal adenocarcinoma’s (PDAC) dismal prognosis is associated with its aggressive biological behavior and resistance to chemotherapy. Epithelial to mesenchymal transition (EMT) has been recognized as a key driver of PDAC progression and development of drug resistance. EMT is a transient and reversible process leading to transdifferentiation of epithelial cells into a more mesenchymal phenotype. It is regulated by multiple signaling pathways that control the activity of a transcription factors network. Activation of EMT in pre-invasive stages of PDAC has been accused for early dissemination. Furthermore, it contributes to the development of intratumoral heterogeneity and drug resistance. This review summarizes the available data regarding signaling networks regulating EMT and describes the integral role of EMT in different aspects of PDAC pathogenesis.

**Abstract:**

Pancreatic ductal adenocarcinoma (PDAC) is one of the deadliest malignancies, characterized by aggressive biological behavior and a lack of response to currently available chemotherapy. Emerging evidence has identified epithelial to mesenchymal transition (EMT) as a key driver of PDAC progression and a central regulator in the development of drug resistance. EMT is a reversible transdifferentiation process controlled by complex interactions between multiple signaling pathways such as TGFb, Wnt, and Notch, which converge to a network of specific transcription factors. Activation of EMT transcriptional reprogramming converts cancer cells of epithelial differentiation into a more mesenchymal phenotypic state. EMT occurrence in pre-invasive pancreatic lesions has been implicated in early PDAC dissemination. Moreover, cancer cell phenotypic plasticity driven by EMT contributes to intratumoral heterogeneity and drug tolerance and is mechanistically associated with the emergence of cells exhibiting cancer stem cells (CSCs) phenotype. In this review we summarize the available data on the signaling cascades regulating EMT and the molecular isnteractions between pancreatic cancer and stromal cells that activate them. In addition, we provide a link between EMT, tumor progression, and chemoresistance in PDAC.

## 1. Introduction

Pancreatic cancer, one of the deadliest solid malignancies, was the seventh leading cause of cancer related deaths worldwide in 2020 [1] and has the lowest 5-year survival rate (about 9%) compared to any other cancer subtype [2,3]. Pancreatic ductal adenocarcinoma, which comprises about 90% of pancreatic cancer cases [4], is characterized by aggressive biological behavior and an enhanced invasive and metastatic potential. Its rapid progression, in addition to the lack of early clinical manifestations, leads to the diagnosis of the majority of cases (about 80%) at an advanced stage, when the available therapeutic options, such as surgical resection and chemotherapy, offer no significant survival benefit [5,6,7,8]. Lack of response to the most commonly used chemotherapeutic regimens, including FOLFIRINOX and gemcitabine-paclitaxel, is suggested to be mediated by both cancer-cell intrinsic [9,10] and tumor microenvironment (TME)-dependent mechanisms [11,12,13,14].

**Figure 1:**
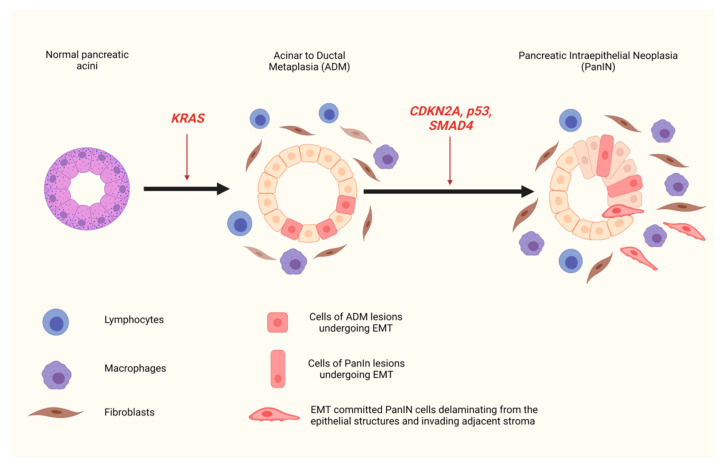
EMT and early dissemination. Activation of EMT in pre-cancerous lesions of pancreatic ductal adenocarcinoma is triggered by the inflammatory microenvironment. A number of cells within acinar-to-ductal metaplasia and Pancreatic Intraepithelial Neoplasia lesions undergo EMT. Some of the EMT-committed cells of PanIN delaminate from the ductal structures and invade the adjacent stroma, before the formation of an invasive tumor.

In the last few years, large scale genomic and transcriptomic studies in human PDAC samples, as well as in vivo experimental studies in genetically engineered mouse models, have led to the characterization of the main genetic alterations associated with PDAC and to a better understanding of the evolutionary trajectory of the disease progression. Carcinogenesis of PDAC is driven by progressive accumulation of mutations in multiple genes, among which the most important are the oncogene *KRAS* and the tumor suppressor genes *p53*, *CDKN2A*, and *SMAD4* [15,16]. These genetic perturbations are accompanied by specific morphological changes that represent different stages of tumor progression. In the majority of cases, the first step of those changes is believed to be a process of acinar-to-ductal metaplasia (ADM), during which acinar/centroacinar cells transdifferentiate into duct-like cells [17,18,19]. This transdifferentiation process is followed by the formation of precursor lesions, termed as Pancreatic Intraepithelial Neoplasia (PanIN), with increasing histologic grades of dysplasia, comprising cuboidal to columnar epithelial cells with aggravation in cellular atypia and architectural distortion (Figure 1). The next step of high grade PanIN is the development of invasive carcinoma [20,21]. Signature genetic alterations occurring in the PDAC tumorigenesis process that drive progression of pre-invasive lesions (ADM, low grade-PanIN, high grade-PanIN) to invasive disease are depicted in Table 1. This progression from normal histology to pre-invasive lesions and invasive tumors is accompanied by the formation of a desmoplastic and fibroinflammatory microenvironment, characterized by the recruitment of heterogenous stromal and immune cells and the production of a dense extracellular matrix [22,23,24]. Even though accumulated knowledge has provided valuable insight into the molecular changes that promote PDAC evolution, they have failed to translate into new targeted therapeutic options for patients.

There is increasing evidence that epithelial to mesenchymal transition (EMT) plays a pivotal role in PDAC evolution. The present review summarizes current knowledge on the signaling cascades regulating EMT and the molecular interactions between pancreatic cancer and stromal cells. In addition, we provide a link between EMT, tumor progression, and chemoresistance in PDAC.

## 2. Epithelial to Mesenchymal Transition and Cancer

EMT is a transient and reversible transdifferentiation program, activated during embryonic development, tissue repair, and tumorigenesis [25,26,27,28]. It involves multiple genetic and epigenetic alterations that occur in a stepwise manner and lead to the generation of cells in intermediate states along the epithelial to mesenchymal axis. This change of cell phenotype is regulated by a network of specific transcription factors (EMT-TFs), including Slug, Snail, Twist, and Zeb, which act pleiotropically on a number of genes controlling different aspects of cellular physiology in order to progressively repress epithelial features and activate mesenchymal ones. Key molecular switches induced by the EMT transcriptional program include: cytoskeletal remodeling through replacement of epithelial cytokeratins by mesenchymal intermediate filament vimentin, loosening of intercellular junctions between adjacent cells through partial repression of E-cadherin and activation of N-cadherin, and elevated expression of matrix-metalloproteinases. This widespread reprogramming of the gene expression profile encompasses alterations in multiple phenotypic features of epithelial cells, such as changes in cell morphology, from squamous, cuboidal, or columnar shapes to spindle-like forms; displaying loss of apical–basal polarity and concomitant gain of front–rear polarity. Moreover, epithelial cells undergoing EMT acquire motile characteristics and an ability to degrade and reorganize the extracellular matrix [29].

Activation of EMT programming during certain steps of embryonic development is critical for the interconversions and migration of cells that are required for normal tissue morphogenesis. In addition, EMT plays an essential role in maintenance of epithelial homeostasis by orchestrating tissue repair, especially the re-epithelialization phase of wound healing [25,26,27,28]. In the context of malignancy, the acquisition of mesenchymal features driven by EMT enables cancer cells to complete many steps of the invasion-metastasis sequence, such as invasion of surrounding tissues, vascular intravasation, and colonization of distant organs [30,31]. Importantly, the phenotypic switch induced by EMT activation is partial, so that cancer cells rarely lose all of their epithelial characteristics, acquiring a full spectrum of mesenchymal traits. In vivo experiments have provided evidence that EMT transcriptional reprogramming generates different tumor cell populations, which correspond to distinct intermediate states between epithelial and mesenchymal phenotypes, including hybrid subpopulations. These subpopulations are associated with diverse properties regarding their clonogenic capacity, differentiation, invasive, and metastatic potential [27,32,33]. Furthermore, disseminated cancer cells need to re-initiate tumor growth in order to serve as founders of metastatic colonies. Thus, enhanced self-renewal and tumor-initiating potential are pre-requisites for successful metastatic foci development, implying a link between the activation of the EMT program and cancer stem cells theory [34,35]. According to the latter, a small population of neoplastic cells, termed as “cancer stem cells” (CSCs) are able to survive under adverse conditions and reproduce themselves, sustaining continuous tumor growth, partly recapitulating the hierarchical organization processes encountered in normal tissues [36]. Indeed, there is experimental evidence that the mesenchymal-appearing cells generated by EMT acquire a number of features associated with a stem-cell like identity, such as low proliferation rate and enhanced tumor-initiating potential, while they have also been found to express well-accepted CSCs markers [37,38]. In support of the potential interplay between epithelial-mesenchymal plasticity and the acquisition of stem-cell like features, signaling pathways regulating CSCs phenotype and those that induce EMT are characterized by significant overlap, as it will be analyzed in the following sections.

Histopathologically, the hallmark of carcinoma invasion is single cells, small compact groups, or elongated strands of cells that penetrate normal tissue. In routine pathological examination, the most commonly observed invasion unit is a group of cells that maintain their cohesion by retaining expression of intercellular junction molecules [39,40,41]. The cells located at the leading edge of these multicellular units undergo partial and transient EMT and develop “leader cell” traits in order to guide their invasion [42,43,44]. In contrast, single disseminated tumor cells are rarely identified during everyday histological evaluation. The presence of such isolated cells, dissociated from the tumor mass at the invasive front, is termed as “tumor budding”, which has emerged as an independent prognostic factor, associated with aggravated clinicopathological parameters and diminished overall survival across a variety of solid malignancies. Biologically, “tumor budding” has been long hypothesized to be associated with EMT. However, the understanding of the dynamic nature of this process is restricted by the fact that its investigation is performed exclusively on histological samples [45,46]. Consistent with the potential link between EMT and tumor budding, immunohistochemical studies of specimens from a variety of epithelial tumors, including oral squamous cell, esophageal and endometrial carcinoma, as well as colorectal and pancreatic ductal adenocarcinoma, have revealed reduction or complete abolishment of E-cadherin expression. In the cases of oral squamous cell carcinoma and pancreatic ductal adenocarcinoma, the alteration of E-cadherin expression is accompanied by a simultaneous increase in the levels of EMT transcription factors Zeb1 and Zeb2 within tumor buds [47,48,49,50,51,52,53,54].

## 3. Epithelial to Mesenchymal Transition in PDAC

Accumulating data suggest that PDAC lethal behavior is at least partly related to EMT activation. Immunohistochemical studies on resected PDAC specimens have shown altered expression of EMT-TFs in ductal tumor cells compared to surrounding parenchyma [55]. In more detail, Snail exhibited moderate to strong expression, Slug showed less intense staining, and Twist was absent or only weakly expressed. Moreover, N-cadherin expression was detected in cancer cells and was more prominent in infiltrating areas [55]. Additional experiments based on the generation of orthotopic tumors by implantation of multiple human pancreatic cancer cell lines in nude mice revealed predominant Slug expression at the invasive front, whereas Snail was primarily found at the tumor center. The metastatic capacity of tumors was also positively correlated with an undifferentiated phenotype of the cell lines and higher Snail transcription levels. The diversity and regional variability in expression patterns of single EMT-TFs within the tumor is a hallmark feature of most carcinomas. It is almost certainly a consequence of the highly complex nature that defines the heterotypic signaling circuits governing EMT-TFs regulation and highlights the specialized and context-specific role of each member of the transcription factors family [56]. A retrospective study of 174 PDAC patients revealed a strong correlation between high levels of EMT-TFs and the presence of lymph node metastasis (*p* = 0.03) or portal vein invasion (*p* = 0.038) [57]. A recent meta-analysis has also confirmed a vital role of EMT in the appearance of tumor budding (TB) [53,54,58,59,60,61,62] and demonstrated a statistically significant association between high grade TB and increased risk of mortality or disease recurrence. Table 2 summarizes the retrospective studies that have demonstrated a statistically significant association between high grade TB and reduced overall survival and/or disease free survival [58,59,61,63,64,65,66].

Two fundamentally different metastatic cell models have been suggested. According to the classical one, metastasis is the final step of a “Darwinian” evolutionary process. This model states that metastatic competent clones emerge in the context of the primary tumor, after multiple successive cycles of genetic and epigenetic changes, followed by selection pressure [67]. The second model envisions metastasis as an inherent property of tumors, arising very early in their natural history. It supports that cellular dissemination can occur before the formation of a histologically identifiable invasive carcinoma [68,69], and it is consistent with recent studies that revealed the presence of tumor cells in the bone marrow of patients with in situ mammary neoplasia [70]. This second metastasis model is supported by novel in vivo studies on two of the most frequently used genetically engineered PDAC mouse strains, which are based on simultaneous conditional KRAS gain-of-function mutation and *p53* (PKC) [71] or *p16* (IKC) [22] deletion. Lineage-tracing experiments on both those mouse strains, which fully recapitulate tumor histology, desmoplastic stroma, and metastatic spread in humans, revealed the expression of EMT transcription factor Zeb1 in areas of acinar-to-ductal metaplasia (ADM) as well as in cells of pre-invasive lesions of low and high grade PanIN [72]. Moreover, single Zeb1+ tumor cells with a spindle-like morphology that had delaminated from PanIN were identified in adjacent stroma and also in the circulation and the liver of mice without frank pancreatic tumor development (Figure 1). Similar findings were encountered on human PDAC. Interestingly, the EMT-committed disseminated cells displayed stem-like properties, such as an enhanced tumor initiating capacity associated with an increased potential of metastatic colonies formation in distant organs [72]. The above data point towards a link between EMT activation and early cancer cell dissemination. It should be noted that this hypothesis comes in contrast with previous studies supporting that metastatic clones appear at an advanced stage of pancreatic cancer evolution [73]. A plausible explanation could be that cancer cell spreading occurs years before metastatic foci become clinically evident.

Furthermore, in the study of Rhim et al. [72], cerulein and duct ligation induced pancreatitis accelerated the emergence of ADM and PanIN in PKC and IKC mouse strains, and led to a higher proportion of cells undergoing EMT, invading the surrounding tissue, and entering the bloodstream. This finding confirms the well-established role of inflammation in promoting tumor development and provides evidence that a pro-tumorigenic inflammatory microenvironment is a key driver of the EMT-regulated early carcinoma invasion and dissemination process [72,74,75]. In addition, cells of pre-invasive lesions have been shown to act as inflammatory triggers, promoting stromal reaction and surrounding tissue infiltration by multiple fibroblasts and immunoregulatory cells. These heterogenous populations of the microenvironment secrete a variety of growth factors and cytokines, which act as paracrine signals on epithelial neoplastic cells and activate multiple pathways that converge to the EMT-transcription factors, enabling progression of pre-invasive lesions to invasive carcinoma [76] (Figure 1).

EMT appears to be equally important during all stages of tumors progression by being a key driver of cellular plasticity and intratumoral heterogeneity. Recently, implementation of single cell RNA sequencing technology provided a more comprehensive and higher resolution analysis of the full spectrum of different tumor cell populations encountered in pancreatic cancer. Single cell analyses of tumors derived from PKC mouse models, as well as from human PDAC surgical specimens, identified multiple distinct phenotypic cancer cell clusters, which were classified into categories along the epithelial-to-mesenchymal continuum based on their gene expression profile. The epithelial clusters showed enrichment for genes linked to epithelial differentiation and proliferation, whereas the mesenchymal exhibited an EMT-related profile. There were also many hybrid populations with intermediate phenotypes and gene signatures, which corresponded to a wide range of states within the EMT spectrum. The EMT program is therefore heterogenous on a single cell level permitting continuous fluctuations among different cancer cell populations, contributing to tumor heterogeneity. There also seems to be a strong correlation between the stage of tumorigenesis and the dominant cell population phenotype. In early stages, tumors displayed a predominant epithelial profile, whereas cancer cells enriched for mesenchymal markers seemed to emerge in more advanced stages of tumor evolution [77]. The dynamic interconversions among different phenotypic states could explain the pivotal role of EMT to the emergence of cancer cells with stemness features, namely CSCs. Since their first identification in 2007 [78], a broad spectrum of markers and functional in vitro and in vivo assays, such as anchorage independent growth (tumor spheres) and limiting dilution xenograft models in immunodeficient mice, have been utilized for the prospective isolation and characterization of CSCs in pancreatic carcinomas [79,80,81,82,83,84]. The most promising candidate markers utilized so far are CD44, CD24, EpCAM (ESA), ALDH, CD133, and c-Met. Those markers have been used for the identification and isolation of tumor cells, principally from mouse xenografts of human pancreatic adenocarcinomas and in the case of CD133 also directly from patient specimens. The isolated cells demonstrated significantly enhanced tumor initiating capacity when injected in immunodeficient mice, as well as higher potential in generating tumor spheres in vitro [78,79,80,81,82,83,84]. Even though a common signature for their detection is still missing, there is evidence that activation of EMT induces expression of CSCs markers, reflecting the potential interplay between epithelial-to-mesenchymal plasticity and CSCs.

Intratumoral heterogeneity of PDAC is directly associated with the spatial heterogeneity of the tumor microenvironment (TME). Regional variations of both its cellular and non-cellular constituents create a mosaic that modulates intratumoral phenotypic patterns. Cancer associated fibroblasts (CAFs) constitute the most abundant cellular component of the tumor stroma, engulfing different subpopulations characterized by molecular, immunophenotypic, and functional divergence. Through a dynamic network of interactions, CAFs execute either tumor suppressive or tumor restrictive functions and actively induce phenotypic shifts in cancer cells [85,86,87,88]. For example, the genetic depletion of *SMA+* cancer associated fibroblasts in a KRAS-driven PDAC mouse model led to reduced production of collagen I in tumor stroma and to the generation of tumors with an anaplastic phenotype, accompanied by the acquisition of an EMT signature and an enrichment of cells expressing CSCs markers [89]. Results from co-culture experiments of PDAC cell lines and cancer associated fibroblasts (CAFs) revealed a strong association between CAF concentrations and tumor cells gene expression profile. In more detail, low CAF concentration correlated with a pure, either proliferative or EMT tumor cell signature, whereas CAF enrichment led to a mixed, proliferative and EMT profile [90]. The TME of PDAC is also highly rich in leukocytes, encompassing a broad spectrum of both innate and adaptive immunity cells [23]. Those heterogeneous cell populations are engaged in an elaborate network of provisional interactions among themselves and the neoplastic cells, directly influencing their phenotypic plasticity. Two predominant cellular components of the inflammatory infiltrate are tumor associated macrophages (TAMs) and T-regulatory cells (T-regs), that work in a co-operative manner to establish an immunosuppressive niche and undermine the development of an effective tumoricidal immune response [91,92,93]. They also secrete inflammatory cytokines and other paracrine factors such as interleukin-1, Matrix metallopeptidase 9, or Toll-like receptor-4 agonists or interact with tumor cells in a juxtracrine manner, for example through CD90, acting as potent inducers of EMT [94,95,96,97,98,99]. This direct effect postulates a mechanistic link between immunosuppression and enhanced invasive capability of pancreatic tumor cells.

The above data suggest that in early stages, TME-epithelial interactions promote EMT leading to initiation of invasion and cell dissemination, whereas in more advanced disease a more complex interplay is encountered between the different TME subpopulations and cancer cells.

## 4. Pathways Regulating Epithelial to Mesenchymal Transition in PDAC

**Figure 2:**
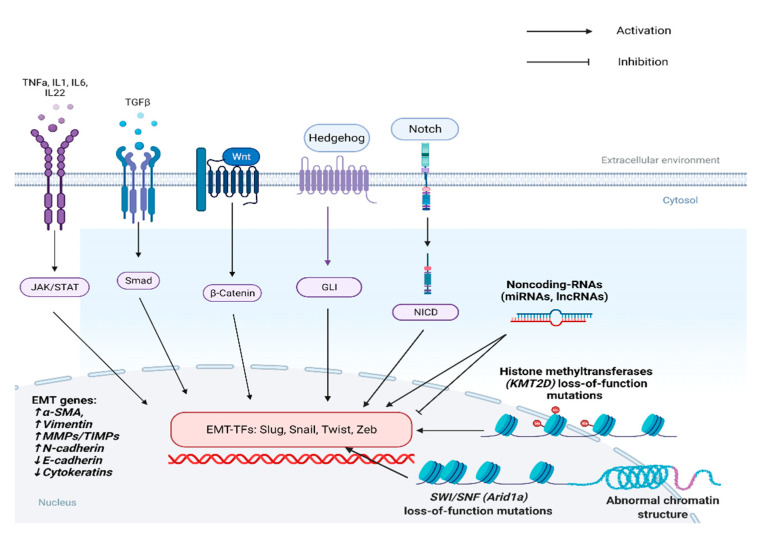
Multiple signaling pathways and epigenetics factors, including histone methyltransferases, chromatin remodeling complexes, and noncoding-RNA molecules control EMT in PDAC. (a-SMA: alpha Smooth Muscle Actin, MMPs: Matrix Metalloproteinases, TIMPs: Tissue Inhibitors of Metalloproteinases).

The broad reprogramming of the gene expression profile that occurs during EMT requires the synergistic activity of many different paracrine factors and the coordinated modification of multiple signaling pathways. Many cytokines, growth factors, metabolic regulators, post-translational and epigenetic modifiers, components of the DNA damage response machinery and differentiation factors have been identified as key players of EMT transcriptional reprogramming (Figure 2). In the following section, the prevailing pathways and mediators regulating EMT transition in PDAC are analyzed.

Inflammatory cytokines: Among the various cytokines produced by CAFs and immune cells of the TME, the most well-characterized inducers of EMT are TGF-β, IL6, IL1, TNFa [76], and IL22 [100].

Transforming growth factor-β (TGF-β): TGF-β is the major EMT-activator in a number of different cancer subtypes, including PDAC [101]. It acts mainly through engagement of TGF-β receptor, which leads to phosphorylation of *SMAD2* and *SMAD4*. Those two transcription factors subsequently form heterodimers, which translocate to the nucleus and drive or repress transcription of several genes [102]. Its role in pancreatic cancer progression is cellular context dependent. In cancer cells, induction of TGF-b β pathway directly activates transcription of Slug, Snail, Twist, and Zeb1 leading to EMT initiation, which both reduces proliferation by cell-cycle arrest but also promotes invasion [103,104]. Moreover, stromal TGF-β signaling promotes tumor growth by inducing fibrosis and creating an immune suppressive microenvironment [105].

Interleukin-6 (IL6): IL6 has been found to play a critical role in KRAS-induced malignant transformation in the pancreas [106]. Its oncogenic function is executed through ligation of IL6-Receptor, which results in phosphorylation of JAK kinases and subsequent phospho-activation of the *STAT3* transcription factor. *STAT3* then translocates to the nucleus and acts as a master regulator of pancreatic tumorigenesis in multiple stages of cancer progression [107]. Genetic deletion of *STAT3* in genetically engineered mouse models has been shown to reduce inflammation-driven progression of PanIN to invasive PDAC [108,109]. Moreover, *STAT3 CRISPR/Cas* mediated ablation in murine *KRAS/p53* null cells led to the formation of xenografts with anaplastic morphological and immunophenotypical characteristics, including loss of E-cadherin and keratin expression with subsequent acquisition of SMA positivity, manifesting EMT-activation [110]. In addition, *STAT3* has been mechanistically shown to bind to regulatory sequence of vimentin gene directly inducing its expression, further supporting the key role of *STAT3* and IL-6 in EMT [111].

Interleukin-1 (IL1) and tumor necrosis factor-a (TNF*a*): IL1 and TNFa are two proinflammatory cytokines that execute their function through activation of Nfk-b transcription factor. The latter is constitutively active in the majority of PDAC and is associated with poor prognosis [112]. It regulates a wide spectrum of biological processes, including proliferation, apoptosis, and inflammation. Recent evidence suggests that in addition to those well-established functions, Nfk-b is also a central regulator of tumor cells metastatic potential. Its activation by IL-1 and TNFa signaling axes induces a mesenchymal phenotype of pancreatic cancer cells and leads to enhanced migratory capacity, whereas its inhibition substantially reduces their invasive properties. These phenotypic alterations are, as expected, associated with upregulation of vimentin and *Zeb1* and downregulation of *E-cadherin* [113,114,115].

Interleukin-22 (IL22): IL22 has been recently identified as a pro-tumorigenic cytokine in pancreatic cancer. Its expression is upregulated in human PDAC samples, and its role has emerged as a key mediator of the crosstalk between tumor and immune cells in early steps of tumor development. Namely, IL22 promotes acinar-to-ductal metaplasia and induces expression of EMT in KPC mice. These oncogenic effects appear to be *STAT3*-dependent as they were alleviated by its pharmacological inhibition [100].

Cancer Stem Cells pathways: Wnt, Notch, Hedgehog, and Hippo are evolutionarily conserved signaling pathways, implicated in multiple cellular processes, such as proliferation, differentiation, and stem cell renewal. In many organs, including the pancreas, they act as crucial regulators of both embryonic development and adult tissue homeostasis [116,117,118,119,120,121,122,123,124,125,126,127]. Aberrant activity of these pathways in different malignancies underpins a synergy in tumor growth. Their functional relevance is mainly associated with the establishment and maintenance of a cancer stem cell phenotype and the promotion of tumor invasion and metastasis [128,129,130,131,132,133]. Abnormal activity is a common trait of PDAC, and experimental data derived mainly from in vitro studies propose a leading role in the induction of an EMT transcriptional program associated with acquisition of a mesenchymal phenotype, enhanced invasive and migratory potential, as well as expression of cancer stem cells markers.

Wnt pathway mediates multiple biological processes depending exclusively on b-catenin for signal transduction. B-catenin protein levels are regulated at a post-translational level by a complex network of modifiers [134]. Foxo3a and SPRY2 act as negative modulators of the Wnt pathway by inhibiting b-catenin-mediated cellular responses. Their knockdown in pancreatic cancer cell lines enhances b-catenin activity, induces EMT, and promotes metastasis [135]. In contrast, Ataxia Telangectasia group D Complement gene (ATDC), a positive regulator of b-catenin, has been shown to activate master EMT transcription factors as well as cancer stem cells markers. ATDC is overexpressed in the vast majority of human PDAC, whereas its conditional knockout in a KRAS induced pancreatic tumorigenesis murine model completely abrogated invasive cancer development. In addition, in vivo studies from the same group showed that ATDC conditional overexpression in the context of activating KRAS mutations resulted in acceleration of tumorigenesis and increased metastatic burden [136].

Notch is a juxtracrine signaling system that relies on interactions between adjacent cells, leading to direct activation of two families of transcription factors: Hey and Hes [137]. It is a critical pathway for stem cell renewal and cellular differentiation that exerts both oncogenic and onco-suppressive functions depending on the tumoral context [138,139]. In PDAC, Notch role is oncogenic, promoting EMT [140]. Pharmacological inhibition in pancreatic cancer cell lines led to diminished EMT activation and reduced expression of CSCs markers [141]. Recently, an interesting indirect role of Notch in promoting EMT and metastasis of pancreatic cancer was described, based on an inflammatory feedback circuit between tumor cells and macrophages. Autocrine activation of Notch in cancer cells stimulates the secretion of cytokines that induce recruitment of macrophages and their deviation towards a tumor-supporting M2 phenotype, also termed tumor associated macrophages (TAMs). TAMs then activate EMT, initiating tumor invasion [142].

Hedgehog signaling system exerts a multitude of functions in both tissue homeostasis and cancer development. Activation of the pathway incites a complex intracellular cascade that begins from the transmembrane protein SMO and terminates at the intranuclear transactivators GLI1/GLI2 [143]. In PDAC pathogenesis, Hedgehog’s multifaceted role includes tumor cell-intrinsic actions [144], and it also seems to mediate interactions between neoplastic cells and the various cellular components of TME [145]. In vitro experiments in pancreatic cancer tumor spheres indicate a pro-tumorigenic function of the Hedgehog pathway, through EMT induction and regulation of cancer stem cells plasticity. Knockdown of SMO resulted in reduced expression of EMT markers and diminished invasive potential of pancreatic cancer stem cells [146].

Hippo pathway regulates various biological processes by tightly controlling the activity of transcriptional co-activators YAP/TAZ. Stimulation of the pathway induces a phosphorylation cascade of multiple intracellular mediators (MST1/2, LATS1/2) that ends up in the inactivation of YAP/TAZ, whereas inhibition triggers their translocation to the nucleus, enabling them to transactivate a wide range of downstream target genes involved in both cellular physiology and carcinogenesis [147]. In pancreatic cancer there is data supporting an oncogenic, EMT promoting, function of *YAP/TAZ*. *YAP* expression is elevated in human PDAC specimens, whereas induced overexpression and silencing of both *YAP* and *TAZ* in PDAC cell lines indicate their functional importance in EMT induction accompanied by an enhanced invasive and migratory capacity [148,149].

Epigenetic regulation of EMT: Epigenetic reprogramming plays a significant role in modulating PDAC progression, partly by regulating the EMT transdifferentiation program. Frequent alterations in chromatin remodelers, histone methyltransferases, and noncoding RNAs are a hallmark feature in a significant proportion of human pancreatic tumors.

The SWI/S*NF* is a chromatin remodeling complex that regulates gene expression by controlling transcription factors access to regulatory DNA sequences, especially enhancers. One of its critical components, Arid1a, is a vital regulator of pancreatic cell fate and an essential factor for acinar cell differentiation and homeostasis [150]. Its key role in cancer biology is underpinned by the frequent loss of function mutations in multiple malignant tumors, including PDAC, where it is found mutated in about 4% of cases [151]. Arid1a inactivation is correlated to worse prognosis and poor tumor differentiation and its conditional ablation in a *KRAS*-driven PDAC murine model accelerated significantly invasive tumors formation, a finding underlying its tumor suppressor potential. Mechanistically the reduced tumor latency seemed to be linked to the elevated protein levels of Myc [152]. Furthermore, *Arid1a* knockdown in pancreatic cancer cell lines induced a phenotypic shift to a more mesenchymal state, associated with elevated expression of EMT related markers, such as vimentin and *N-cadherin* [153].

KMT2D is another chromatin modification enzyme that acts as a major histone methyltransferase establishing active promoter and enhancer landscapes by mono- and di-methylating lysine 4 residues in histone 3. Its expression has been found to be suppressed in human PDAC specimens, whereas its CRISPR/Cas based ablation in pancreatic cancer cell lines lead to the generation of cells with a mesenchymal-spindle like phenotype along with an enhanced migratory and invasive capacity and enrichment of an EMT signature. In addition, the KMT2D-null cells generated tumors with more mesenchymal cell morphology in orthotopic xenografts experiments [154].

Micro-RNAs (miRNAs) and long non-coding-RNAs (lncRNAs) are RNA molecules that simultaneously control the expression of multiple genes at a post-transcriptional and posttranslational level. Their aberrant expression is a landmark feature of malignant tumors, underlying their profound contribution to the progressive and dynamic deregulation of various biological processes that define cancer cells, including proliferation, apoptosis, invasion, and metastasis [155,156]. Both miRNAs and lncRNAs can function as either oncogenes or tumor suppressor genes in a wide range of neoplasms [155,156] including PDAC [157,158]. The bulk of data regarding the multipronged functions of non-coding RNAs in pancreatic carcinogenesis have been derived from in vitro experiments, based on inducible overexpression or genetic deletion of distinct RNA molecules in different PDAC cell lines followed by evaluation of this interference in the expression levels of downstream target genes and the phenotypic plasticity of tumor cells. This strategy has accentuated the notable role of non-coding RNAs in regulating EMT and has identified a broad spectrum of RNA molecules that either enhance or impair tumor cells’ invasive and metastatic capacity. Namely, such an experimental approach has elucidated a negative feedback loop between Zeb1 and two members of the miRNA-200 family (miRNA-200c and miRNA-141) that triggers the invasive potential of pancreatic cancer cells [159,160]. MiRNA-200c and miRNA-141 are downregulated in pancreatic cancer cell lines and act in an EMT-inhibitory manner being engaged in reciprocal suppressive interactions with Zeb1. Both miRNA molecules restrain Zeb1 expression and vice versa. Consequently, Zeb1 upregulation impairs miRNA-200c and miRNA-141 transcription and stabilizes an EMT phenotype in tumor cells [159,160]. MiRNA-34b, miRNA-126, miRNA-146, miRNA-203, and miRNA-218 inhibit phenotypic shift of pancreatic cancer cells to a mesenchymal state and compromise their invasive capacity [161,162,163,164,165]. In contrast, miRNA-10a and miRNA-208 enhance cells’ metastatic behavior [166,167,168]. In a similar fashion, lncRNAs employ more complex mechanisms to control a broad spectrum of downstream target genes and act as positive or negative modulators of cellular invasiveness. SNHG1, DLEU2, HULC2, XIST, and PVT1 are the most well-characterized lncRNAs displaying an EMT promoting activity [169,170,171,172,173], whereas ENST00000480739, PCTST, and ZEB2-AS1 have been identified as potent inhibitors of pancreatic tumor cells invasive capacity [174,175,176]. Table 3 summarizes the studies on human PDAC specimens addressing the expression levels of non-coding RNA molecules. When available, correlation with patients’ prognosis is also depicted.

Miscellaneous pathways: GATA6 expression is one of the key factors regulating epithelial differentiation during pancreatic embryonal development, and its role in PDAC has recently begun to be uncovered. Pancreatic carcinomas displaying high levels of GATA6 are associated with better tumor differentiation and improved patient outcomes. On the contrary, PDAC cases characterized by loss of GATA6 expression show concomitant activation of epithelial to mesenchymal transition, acquisition of an EMT molecular phenotype, and poor prognosis. Gain and loss of function experiments on PDAC cell lines confirmed the role of GATA6 in regulating EMT [177]. According to a recent study, GATA6 levels are, at least partly, controlled by an epigenetic mechanism, based on EZH2-induced transcriptional repression [178].

DNA damage response (DDR) acts as a barrier that prevents progression of intraepithelial lesions to invasive cancer, whereas defects of DDR pathways lead to EMT activation. Ataxia telangectasia mutated (ATM) factor deletion in the context of KRAS activating mutations enhance the development of pre-cancerous lesions such as acinar-to-ductal metaplasia associated with expression of EMT factors. Moreover, tumors developed in an ATM null background exhibit an enrichment of a CSC-marker positive population [179].

PP2A-SET interplay is speculated to have an integral role in EMT. SET is an endogenous inhibitor of tumor suppressor PP2A and has been found overexpressed in multiple pancreatic cancer cell lines. It executes pro-tumoral activity through PP2A-downregulation and c-Myc stabilization. It also triggers EMT-transcriptional factors leading to acquisition of a mesenchymal phenotype and to increased invasive and migratory potential [180]. CIP2A is another negative regulator of PP2A activity that has been statistically associated with high expression of EMT transcription modulators in pancreatic cancer. However, no mechanistical data exist that could suggest a causal relationship [181].

Metabolic plasticity has been also linked to EMT activation on tumor cells. FOXM1 is a transcription factor modulating EMT in multiple carcinomas, including PDAC, where it seems to acts as an upstream regulator of Snail, promoting EMT in a glucose dependent manner [182]. In another study employing a PDAC orthotopic mouse model, glutamine deprivation led to overexpression of Slug, a proliferation repressor and EMT inducer, resulting in reduction of tumor metabolic demands. This nutrient-stress Slug-mediated reprogramming confers a cell survival advantage and fosters metastasis [183]. Further experimental evidence is needed in order to elucidate the potential causal link between EMT and metabolic rewiring in PDAC.

## 5. Epithelial-to-Mesenchymal Transition and Chemoresistance in Pancreatic Ductal Adenocarcinoma

Poor prognosis of PDAC is to a large extent associated with lack of response to existing systemic treatments. Recent evidence supports the notion that development of drug resistance is a multi-factorial process and EMT appears to be one of the key factors. In this context, a recent immunohistochemical study on human pancreatic cancer specimens showed elevated expression of EMT markers and increased levels of tumor budding after neoadjuvant chemotherapy [184]. Similar findings have been extracted from in vitro experiments in both cell lines and patient-derived organoids. Namely, Gemcitabine resistant stable PDAC cells lines displayed increased levels of EMT transcription factors and reduced expression of E-cadherin [185,186]. FOLFIRINOX treatment experiments in both commercial and primary patient-derived PDAC cell lines with diverse phenotypes along the epithelial to mesenchymal axis have shown that the quasi-mesenchymal phenotype is related to treatment resistance. Pre-treatment single cell analysis depicted the heterogeneity of cell line population with some cells showing epithelial signatures and others showing mesenchymal signatures. Post-treatment analysis demonstrated significant alterations in the proportion of epithelial to mesenchymal-like cells, with a shift towards an overall prevailing mesenchymal phenotype [187]. In another experiment, Hiriac et al. [188] used patient derived pancreatic cancer organoids, aiming to identify gene expression profiles that predict the level of response or resistance to the standard-of-care chemotherapeutic regimens. In concordance, more resistant organoids displayed an EMT-associated signature [188].

**Figure 3:**
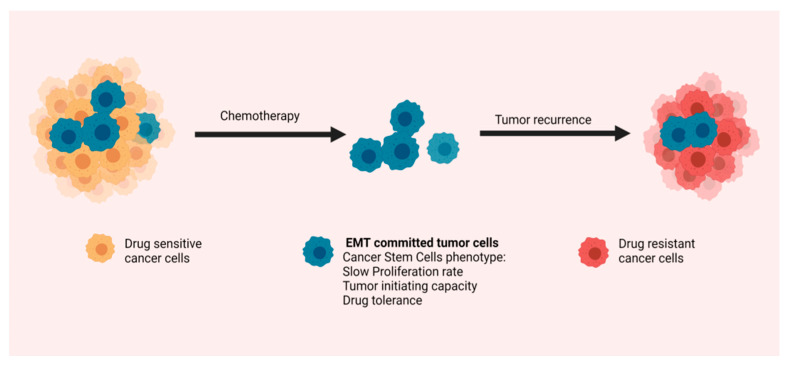
EMT and chemoresistance: Tumor cells undergoing EMT acquire a number of features associated with a cancer stem cell phenotype, which make them intrinsically tolerant to chemotherapy. They are therefore capable of surviving drug exposure and form a new tumor, leading to disease recurrence.

According to the two main theories, chemoresistance development is driven either by preexisting resistant clones or by a small subset of tumor cells that evade chemotherapy, survive, and eventually form a new therapy-resistant tumor. The drug-tolerant cancer cells harbor features of stemness, which largely overlap with CSCs traits, such as slow proliferation rate, plasticity, self-renewal ability, and tumor-initiating capacity. Indeed, CSCs identified in various tumor subtypes, including PDAC, have been found intrinsically more resistant to conventional chemotherapy than their non-CSCs counterparts [35,189,190,191,192] (Figure 3). It is therefore plausible to speculate that the fundamental role of EMT in drug resistance is at least partly interceded by induction of a stem-like identity in tumor cells. In support of this theory, emerging evidence suggests that pancreatic CSCs and EMT mechanistic parallels coalesce on signaling pathways linked to evasion of chemotherapeutic regimens. ATDC represents a good example of such a pathway, with a well-defined multifaceted role in PDAC biology, that has emerged as a critical orchestrator of drug resistance. Its multipronged functions in pancreatic cancer progression include, as already described in the previous section, the activation of EMT, the promotion of migration and metastasis, and a phenotypic shift to a CSCs state. Recently a novel role of ATDC as a crucial modulator of an NRF2-dependent chemoresistance mechanism was revealed [193]. NRF2 is one of the critical components of the cell detoxification machinery and a master regulator of chemoresistance in various tumor subtypes [194,195]. Its levels in PDAC are increased and they seem to be controlled predominantly at a posttranslational level by ATDC. ATDC overexpression in pancreatic cancer cell lines conferred resistance to gemcitabine, whereas NRF2 shRNA-mediated silencing alleviated this effect. These data support the existence of an ATDC/NRF2 axis that controls response of PDAC to gemcitabine [193]. A number of other pathways and factors, implicated in EMT activation, such as Notch [140], YAP [148], and GATA6 [177], have also been shown to confer resistance to the most frequently used chemotherapy regimens, even though specific mechanistic details have yet to be elucidated. EMT mediators have been also found to alter the absorption profile of drugs by regulating the expression of drug influx and efflux transporters. For example, EMT activation is accompanied by loss of gemcitabine chemosensitivity through an equilibrative nucleoside transporter 1 (ENT1) regulated mechanism. Cadherin switching from the epithelial (E) to neuronal (N) type and loss of EpCAM expression reduced ENT1 expression, inhibited its membrane localization, and diminished gemcitabine transport in EMT-committed tumor cells [196].

## 6. Pharmacological Targeting of Epithelial to Mesenchymal Transition

Due to its integral and multifaceted role in pancreatic cancer biology, targeting EMT seems as a feasible therapeutic strategy for PDAC. The abundance of different extracellular mediators and heterotypic intracellular signaling circuits that control EMT means that different stages of this multistep transdifferentiation process can be exploited pharmaceutically. It should, however, be stressed that the intricate networks controlling EMT are also critical modulators of other aspects of pancreatic cancer pathogenesis, and their utilization as therapeutic targets should be carefully designed. TGF-b, IL-6, IL-1, and Hedgehog are among the most potent and “druggable” inducers of EMT, and a multiplicity of developed inhibitors targeting these pathways are in the process of safety and efficacy evaluation in clinical trials of different phases. Table 4 shows the multiple inhibitors and monoclonal antibodies targeting EMT under clinical trials for PDAC [197].

TGF-b: A variety of drugs belonging to three different classes with diverse mechanisms of action have been developed to target the TGF-β pathway, engulfing antibodies directed against TGF-b ligands or receptors (TGF-bRI), inhibitors of TGF-bRI kinase, and antisense oligonucleotides that target TGF-b transcripts. The efficacy of these drugs as first or second line therapeutic options is being assessed in various clinical trials or preclinical models of pancreatic cancer, almost exclusively in combination schemes with common chemotherapy drugs or immunotherapeutic monoclonal antibodies [198,199,200].

IL-6/IL-1: Monoclonal antibodies targeting IL-6 or IL-6 receptors as well as a selective estrogen receptor modulator [201] have been utilized for the inhibition of IL-6-induced EMT in PDAC. Clinical trials of IL-6 pathway inhibitors in combination with chemotherapeutic or immunotherapeutic drugs are currently in progress. The anti-IL-1b mAb canakinumab (ACZ885) is also under evaluation in a phase Ib study in metastatic pancreatic cancer patients.

Hedgehog: Vismodegib is an inhibitor of hedgehog ligand membrane receptors and has been evaluated in a combination scheme with gemcitabine and nab-paclitaxel in a phase II clinical trial. However, the study did not reveal improved efficacy compared to chemotherapy alone [202]. A number of other hedgehog inhibitors are currently in clinical trials for advanced PDAC.

## 7. Conclusions—Future Directions

In the last few years, rigorous research has led to enormous progress in clarifying the contribution of EMT in PDAC pathogenesis. The present review summarizes current knowledge regarding the multifaceted role of EMT in pancreatic carcinogenesis. EMT activation in pancreatic ductal epithelium of precancerous lesions has been linked to early cancer cell invasion and dissemination, stimulated by inflammatory microenvironment. Additionally, EMT has been shown to enhance cellular plasticity and intratumoral heterogeneity and to promote drug resistance, possibly through CSC development. Various signaling pathways that control epithelial to mesenchymal plasticity have been identified, including inflammatory cytokines, CSC pathways, epigenetic modulators, and various others signaling networks. However much work still needs to be done in order to acquire a more comprehensive understanding of the complex non-linear interactions of intracellular signaling circuits that govern EMT and how this transdifferentiation program affects various aspects of pancreatic cancer biology. Even though EMT master regulators have been identified, significant information is missing regarding the exact specialized role that each of these EMT-TFs exerts in the broad rewiring of the gene expression profile and the progressive phenotypic shift induced in tumor cells. Moreover, there is no compelling evidence to provide a mechanistic correlation between activation of EMT and generation of cancer stem cells. It is therefore not yet clarified how the dynamic modification of multiple cellular function induced during epithelial to mesenchymal transdifferentiation is coupled to the acquirement of stemness features, and consequently to the development of drug resistance. The development of effective genetically engineered murine models is urgently needed for a more in depth study of EMT. Additionally, the implementation of the latest state of the art techniques, such as in vivo lineage-tracing, organoids, and gene editing based on CRISPR/Cas, can be proven invaluable tools in the effort to decipher the contribution of EMT in PDAC natural history. In vivo models of PDAC and organoids should also be employed for the rapid evaluation of multiple candidate drugs aiming to develop more effective therapeutic schemes targeting EMT, with the hope to prolong pancreatic cancer patients’ survival and improve their quality of life.

## Figures and Tables

**Table 1:** Signature genetic alterations in each step of the PDAC tumorigenesis process.

Stage of Tumorigenesis	Gene Affected	Type of Genetic Alteration [References]
Acinar to Ductal Metaplasia	*KRAS*	Constitutive activation (upregulation): gain-of-function mutation (KRAS 12GD) [15,16]
Low grade PanIN	*CDKN2A (Ink4a/Arf)*	Inactivation (downregulation): deletion, loss-of-function mutation [15,16]
High grade PanIN	*TP53*	Inactivation (downregulation): loss-of-function mutation, deletion [15,16]
	*SMAD4*	Inactivation (downregulation): deletion, loss of function mutation [15,16]

**Table 2:** Retrospective studies showing a statistically significant association between high grade tumor budding and reduced survival. HR: hazard ratio; RR: relative risk; CI: confidence interval.

Study (Reference)	Number of Patients	Overall Survival	Disease Free Survival
Chouat et al. [59]	50	HR = 6.09 (95% CI 1.11–33.28), *p* = 0.03	HR = 2,87 (95% CI 1.41–17.51), *p* = 0.02
Liu et al. [61]	46	*p* = 0.01	*p* = 0.001
Karamitopoulou et al. [63]	117	HR = 3.98 (95% CI 2.3–6.9), *p* < 0.0001	*p* = 0.0005
Lohnesi et al. [64]	173	HR = 1.040 (95% CI 1.019–1.061), *p* < 0.001	HR = 1.037 (95% CI 1.017–1.058), *p* < 0.001
O’Connor et al. [65]	613	RR = 1.46 (95% CI 1.13–1.88), *p* = 0.004 HR = 2.65 (95% CI 1.79–3.91), *p* < 0.0001	RR = 1.61 (95% CI 1.05–2.47), *p* = 0.03

**Table 3:** Studies showing the expression levels of non-coding RNA molecules in PDAC patients compared to adjacent normal tissue and their correlation with prognosis. PDAC: pancreatic ductal adenocarcinoma; HR: hazard ratio; CI: confidence interval; NA: not available.

Study [Reference]	Non-Coding RNA Molecule	Number of Patients	Results	OS	DFS
Hamada et al. [162]	miR-126	5	Reduced levels in PDAC compared to adjacent normal tissue.	NA	NA
Xu et al. [170]	lnc-RNA DLEU	178	Increased levels in PDAC compared to adjacent normal tissue. High levels correlate with worse OS.	*p* = 0.036	
Feng et al. [171]	lnc-RNA HULC	36	Increased levels in PDAC compared to adjacent normal tissue.	NA	NA
Sun et al. [172]	Lnc-RNA XIST	30	Increased levels in PDAC compared to adjacent normal tissue.	NA	NA
Wang et al. [175]	Lnc-RNA PCTST	48	Reduced levels in PDAC compared to adjacent normal tissue. High levels correlate with longer OS.	HR = 0.11 (95% 0.02–0.49) *p* = 0.004	NA
Gao et al. [176]	Lnc-RNA Zeb2-AS1	39	Increased levels in PDAC compared to adjacent normal tissue. High levels correlate with worse OS and DFS.	*p* < 0.005	*p* < 0.005

**Table 4:** Inhibitors and monoclonal antibodies targeting EMT, with different mechanisms of action, under clinical trials for PDAC.

Drug Name	Mechanism of Action	Clinical Trial Phase	NCT Registry Number
PF-06952229	Inhibitor of TGF-b receptor (TGF-bRI)	I	NCT03685591
BCA101	Inhibitor of TGF-b/EGFR fusion	I	NCT04429542
SHR-1701	Inhibitor of TGF-b (ligand)	Ib/II	NCT04624217
NIS793	mAb anti-TGF-b (ligand)	I	NCT02947165
		II	NCT04390763
Vactosertib (TEW-7197)	Inhibitor of TGF-bRI kinase	Ib	NCT03666832
		II	NCT04258072
Galunisertib (LY2157299)	Inhibitor of TGF-bRI kinase	Ib	NCT02734160
		Ib	NCT02154646
		Ib/II	NCT01373164
Trabedersen (AP 12009)	Antisense oligonucleotide specific for TGF-b	I	NCT00844064
Tocilizumab	mAb anti-IL6 receptor	II	NCT02767557
		II	NCT04258150
		I/II	NCT03193190
Siltuximab	mAb anti-IL6	I/II	NCT00841191
		Ib/II	NCT04191421
Bazedoxifene	Selective estrogen receptor modulator (SERM)-Inhibitor of IL-6/GP130	-	NCT04812808
Canakinumab (ACZ885)	mAb anti IL-1b	Ib	NCT04581343
LDE225	Hedgehog inhibition	I/II	NCT02358161
		Ib	NCT01485744
NLM-001	Hedgehog inhibition	Ib/IIa	NCT04827953
IPI-926	Hedgehog inhibition	Ib/II	NCT01130142
